# Self-regulated learning: the effect on medical student learning outcomes in a flipped classroom environment

**DOI:** 10.1186/s12909-020-02023-6

**Published:** 2020-03-31

**Authors:** Binbin Zheng, Yining Zhang

**Affiliations:** 1 Office of Medical Education Research and Development at the College of Human Medicine, Michigan State University, 965 Wilson Road, A-214B, East Lansing, MI 48824 USA; 2 Department of Foreign Languages and Literatures, Tsinghua University, Beijing, China

**Keywords:** Self-regulated learning, Flipped classroom, Peer learning, Help-seeking

## Abstract

**Background:**

The flipped-classroom model is increasingly being adopted in competency-based medical education. However, it poses a major challenge to students who have not mastered self-regulated learning strategies. This study explores which self-regulated learning skills affect student learning performance in the first 2 years of medical school at a university in the midwestern United States.

**Methods:**

Survey data were used to assess how 146 first- and second-year medical students’ use of self-regulated learning strategies affected their performance on standardized tests.

**Results:**

Based on the results of regression analysis and content analysis, it was found that the use of peer learning and help-seeking positively affected the performance of first- and second-year students, respectively; whereas the use of rehearsal had a negative effect on student learning outcomes.

**Conclusions:**

The study findings imply that during the transition period from traditional lecture-intensive learning to flipped-classroom learning, promoting peer learning and help-seeking could significantly improve students’ academic achievement.

## Background

The pedagogical model known as the ‘flipped classroom’ has been championed as the next frontier in medical education [1, 2]. It allows students to learn foundational knowledge independently before coming to class. This often takes the form of reading materials or video lectures, and thus leaves class time free for the application of knowledge and for student active participation and engagement in higher-order thinking [3, 4]. However, while the number and quality of studies examining this approach in health education have both been increasing in recent years, the potential of flipped classrooms for improving medical education has not yet been confirmed, with existing research having been primarily focused on pharmacy and nursing [5]. One recent systematic review showed that medical students preferred flipped classrooms, but that the effectiveness of such classrooms remains unclear [6]. Research on flipped classrooms in non-medical education has suggested that students in such settings must have self-regulation skills to achieve academic success [7, 8]. This is because, without these skills, they could fail to comprehend learning materials and/or to strategically utilize learning resources before class, which in turn would likely make them less able to follow and benefit from in-class activities [9].

Self-regulated learning (SRL) refers to “an active, constructive process whereby learners set goals for their learning and then attempt to monitor, regulate, and control their cognition, motivation, and behavior, guided and constrained by their goals and contextual features of the environment” [9]. According to Social-Cognitive Theory, SRL is a cyclical and dynamic process characterized by three phases: *forethought* – task analysis and goal setting before learning; *performance control* – use of cognitive strategies such as rehearsal, elaboration, and organization as well as metacognitive monitoring; and *self-reflection* – students judge and develop reasons for their performance [10]. Different SRL strategies could be potentially beneficial for student performance in different learning contexts [11, 12]. For example, research on first-year medical students undertaking a gross anatomy course found that their use of cognitive strategies such as elaboration (e.g., meaning-enhancing additives, concepts, or generalizations) and critical thinking (e.g., faculty/student questioning, peer teaching) was positively associated with higher academic performance [13]. Similarly, time-management was found to significantly predict students’ achievement in basic science learning [12]. A scoping review of SRL in clinical settings suggested SRL had positive effects on clinical skills development [14]. For example, reflection-in-learning was found to be predictive of medical students’ diagnostic skills [15], and students who were more successful at the venipuncture task tended to have better goal setting, self-monitoring, and reflecting strategies than struggling students [16].

However, scant research has been conducted on the relationship between SRL and learning outcomes in flipped-classroom environments. One of our own studies looked at medical students’ SRL in a flipped classroom learning environment and found that students frequently used strategies in the stages of planning and reflection, but less frequently during the learning or monitoring phase. Our study however, did not associate this with learning outcomes [17]. One study conducted in an elementary math course [9] suggested that SRL strategies in flipped learning could benefit students’ construction of knowledge, and thus further improve learning achievement. Interest in SRL is gaining momentum in the sphere of medical education [11]; yet, little research has focused on which specific SRL strategies might be particularly beneficial to medical students in flipped classrooms, or on such students’ periods of transition from lecture-intensive to flipped learning in the early stage of medical school. This study therefore focuses on exploring two research questions:

1. Which SRL strategies affected student learning outcomes in a flipped classroom environment?
2. How did students perceive the affordances and challenges of learning in the flipped classroom environment?

## Methods

This study was conducted in a large public university’s medical school in the midwestern United States. The medical school’s current curriculum, which incorporates competency-based learning and the flipped-classroom model, has been in place since the Fall semester of 2016. As part of the curriculum, students are organized into different learning societies, including small groups of students, clinical faculty, basic and social societies. Each week, students are required to independently review around 15 h of online readings and videos before class, while classroom-based activities focus largely on small-group discussions using case-based and problem-based learning around patients’ Chief Complaints and Concerns (C3) topics. Our new curriculum is designed especially to provide students with one big integrative course (e.g., HM 552 for first-year medical students in the fall semester), composed of various learning activities, including large group activity, small group discussions, clinical simulation lab, virtual imaging lab, and gross anatomy lab.

At the beginning of the Spring 2018 semester, an online survey was distributed to 380 first- and second-year students, asking about their use of SRL strategies within this new curriculum. Participation in the survey was completely anonymous and voluntary. Motivational Strategies for Learning Questionnaire (MSLQ) [18] has been widely used as an aptitude measure of SRL [10], and was adapted in our study as an instrument used to measure SRL strategies. A total of 56 survey items, covering cognitive strategies (i.e., rehearsal, elaboration, organization, critical thinking), meta-cognition, resource management (i.e., time management, effort regulation, peer learning, and help-seeking), and self-efficacy, were included in the survey. We changed several wordings in MSLQ to accommodate our specific context. For example, “lectures” were changed to “class activities” since our curriculum did not contain any lectures. Another example was “in other class activities such as lecture and discussion,” which was changed to “in other class activities such as discussions, simulations, and anatomy.”

All questions were answered via the same seven-point Likert scale, ranging from “1” (not at all true of me) to “7” (very true of me). Five open-ended questions were also included, to capture students’ perceptions of flipped learning (e.g., “What have you liked best about pre-class learning?”; “Based on your experience so far, what are the challenges you have had with your learning?”). A total of 195 students responded, representing a response rate of 51%. Among these 195 respondents, 49 provided incomplete data and were eliminated from further consideration. This resulted in a final sample size of 146, comprising 70 first-year and 76 s-year students.

In addition, we collected students’ results on the National Board of Medical Examiners (NBME) scaled Comprehensive Basic Science Exam (CBSE) that they took on two occasions: the first at the beginning of the Fall semester of 2017 (before-survey: M = 46.25, SD = 10.9), and the second, at the end of the Spring semester of 2018 (after-survey: M = 58.33, SD = 13.9). Since the sampled first year (i.e., Early Clinical Experience) ended by February 2018 before students moved into intersessions where they focused on particular areas of interests and strengthened their knowledge base of necessary sciences, the score in the Fall semester of 2017 was used as the dependent variable. Scores from the Spring semester of 2018 were used as one of the predictors.

To answer the first research question, multiple regression analysis was used to detect the effects of SRL strategies on students learning outcomes, which was performed using the statistical software *Stata 15*. Each student’s Spring 2018 CBSE score was used as the dependent variable, and their Fall 2017 CBSE scores, controlled for nine SRL strategies (i.e., rehearsal, elaboration, organization, critical thinking, meta-cognition, time management, effort regulation, peer learning, and help-seeking), were included as predictors. To answer the second research question, conventional content analysis [19] – in which coding categories were derived directly from the textual data – were adopted and conducted by the first author to explore common themes in participant perceptions of the affordances and challenges of flipped-classroom learning. The codes were then reviewed by the second author and any disagreements were resolved through discussion. Both authors worked together in selecting appropriate quotations that represented major themes.

## Results

### Self-regulated learning strategies and learning outcome

As can be seen in Table 1, the SRL strategy that respondents reported using most frequently was effort regulation (e.g., “I work hard to do well in this class even if I don’t like what we are doing”; M = 4.93, SD = 1.06). This was closely followed in frequency of use by time management (e.g., “I make sure I keep up with the weekly readings and assignments for this course”; M = 4.91, SD = 0.84) and elaboration (e.g., “I try to relate ideas in the readings to other class activities or clinical experiences whenever possible”; M = 4.78, SD = 1.00). The least frequently used SRL strategy was rehearsal (e.g., “When I study for this class, I practice saying the material to myself over and over”; M = 3.64, SD = 1.12), and the second least frequently used was peer learning (e.g., “When studying for this course, I often try to explain the material to a classmate or a friend”; M = 3.75, SD = 1.47).

**Table 1:** Descriptive statistics of self-regulated learning skills and achievement data

	Mean	SD	Min	Max
Pre-test	46.25	10.93	29.0	77
Post-test	58.33	13.93	0.0	93
**Cognitive strategies**	4.17	.83	2.0	6.3
- Rehearsal	3.64	1.12	1.0	6
- Elaboration	4.78	1.00	2.2	7
- Organization	4.07	1.36	1.0	7
- Critical thinking	4.18	1.04	1.8	7
**Meta-Cognition**	4.46	0.75	2.4	7
**Resource management**	4.48	0.78	2.1	6
- Time management	4.91	0.84	2.1	7
- Effort regulation	4.93	1.06	1.8	7
- Peer learning	3.75	1.47	1.0	7
- Help seeking	4.33	1.28	1.2	7
Self-Efficacy	5.00	1.11	1.9	7
*N*	146			

To explore the relationship between individuals’ learning strategies and their learning outcomes, two regression analyses were conducted, one for first- and the other for second-year students (see Table 2). The results indicated that within both groups, use of the cognitive strategy *rehearsal* had a significantly negative effect on learning growth (*β* = − 4.91, *p* < .001 for first-year students; *β* = − 2.69, *p* < .05 for second-year students).

**Table 2:** Regression analysis of learning strategies on learning outcome among first- and second-year students

	Post-test score	
	First-year students	Second-year students
**Cognitive Strategies**		
- Rehearsal	−4.91^***^	−2.69^*^
	(−3.84)	(− 2.06)
- Elaboration	2.75	−1.24
	(1.72)	(−0.84)
- Organization	0.26	2.04
	(0.22)	(1.60)
- Critical thinking	0.75	−2.10
	(0.77)	(−1.67)
**Meta-Cognition**	−0.87	−0.07
	(−0.50)	(− 0.03)
**Resource Management**		
- Time management	−2.25	1.01
	(−1.33)	(0.57)
- Effort	2.16	−1.06
	(1.84)	(−0.72)
- Peer learning	2.00^*^	−0.19
	(2.42)	(−0.20)
- Help seeking	−2.00	2.39^*^
	(−1.87)	(2.52)
**Pre-test Score**	0.88^***^	1.22^***^
	(4.41)	(7.68)
**Constant**	29.48^*^	6.39
	(2.56)	(0.57)
*N*	70	76
*R*^2^	0.597	0.572
*AIC*	482.59	558.45
*BIC*	511.82	588.75

Control variable: gender and self-efficacy; unstandardized coefficients; *t* statistics in parentheses; ^*^p < 0.05, ^**^p < 0.01, ^***^p < 0.001

Of the other eight SRL strategies, two emerged as beneficial to student learning. Among first-year students, use of peer-learning strategies, such as working with classmates to complete assignments or discussing course materials with a group of other students, significantly predicted learning growth (*β* = 2.00, *p* < .05). The learning growth of second-year students was positively and significantly associated with help-seeking behavior (*β* = 2.39, p < .05); the target of which might be a classmate, a more advanced peer, or a course instructor. None of the other six focal strategies were significant predictors of student learning outcomes.

### Affordances and challenges of flipped-classroom learning

Content analysis of participant responses to the open-ended survey questions supported the quantitative finding reported above; that peer learning and help-seeking were beneficial to student learning outcomes. The most frequently perceived affordance of this new curriculum was its support for co-regulated learning [20], i.e., learning experiences shared among peers. Students argued that working with peers helped them to stay engaged, obtain better explanations of course materials, and ask better questions. Some also pointed out that by learning with peers, they were exposed to alternative perspectives and new ways of thinking. One student mentioned liking “being able to work with my small group since every person has a different way of thinking of things and explaining them. There will be three different analogies/explanations thrown out so that it’ll click for everyone.” Some students also favored changes brought about by the flipped classroom: as one noted, “[i]t’s not just a passive learning environment where we have to listen to the instructor the whole time.”

Respondents also pointed out two other main affordances of flipped-classroom learning: case-based learning and learning autonomy. They found the former helpful for “solidifying their understanding”, “walking through important concepts in specific cases”, and “saying things out loud and thinking through new problems”. With regard to the latter, the self-paced independent-learning aspect of their flipped classrooms enabled them to “arrange life around the schedule”.

However, students also voiced certain challenges arising from the new curriculum, especially during their transition from lecture-based learning in their undergraduate schools to flipped-classroom learning in our medical school. Some argued that too little lecture-based teaching was provided, and that too much independent learning was required; leading them to become easily lost due to the large volume of information available. This was further reflected in some of the challenges mentioned in the responses, such as “staying organized” and “figuring out how to study”.

Another frequently mentioned challenge involved time- and resource management, with several students stating that they found it challenging to balance self-learning, small-group discussion, clinic time, and other responsibilities. Some also felt it was difficult to strike a balance between time spent reviewing past material and keeping up with new material. With regard to resource management, quite a few students mentioned having a hard time dealing with the vast quantities of resources provided for pre-class learning. However, others reported using numerous outside resources that had not been provided in their flipped classrooms to supplement course-provided materials.

## Discussion

By examining medical students’ self-reported SRL strategy use and its relationship to their academic achievement, our study revealed that while using cognitive strategy *rehearsal* was negatively associated with student learning outcomes, use of resource management strategies – peer learning and help-seeking – were positively associated with student learning outcomes, for first- and second-year students, respectively. Students’ responses to open-ended questions further confirmed the affordances of peer learning and help-seeking for learning in a flipped classroom environment.

### Rehearsal as a cognitive strategy during learning

Prior studies conducted in non-medical educational settings have reported that the correlation between rehearsal (e.g., memorization or repeated reading) and academic achievement was either weak or non-significant [21, 22]. In contrast, the present study, found that using rehearsal in flipped-classroom environments negatively impacted medical students’ learning growth. This is perhaps because, by its very nature, this strategy focuses on surface-level learning [22, 23]. However, this finding does not necessarily mean that rehearsal was detrimental to student learning, as it might have drawn learners’ attention during the encoding process, even if it did not help them to make connections within their new knowledge or between their new and prior knowledge [18, 24]. It should also be kept in mind that the current study focused on the integration of basic science and clinical experiences, and thus heavily emphasized the participants’ knowledge connections and applications, rather than their memorization skills. In addition, prior researchers have suggested that the flipped classroom itself favors problem-solving skills and higher-level cognitive skills, such as applying, analyzing, and evaluating, over lower-level ones, such as memorization and information recall, which rehearsal might be more helpful in improving [25]. Thus, it is not particularly surprising to find a negative impact of rehearsal on learning outcomes in this case.

### Develop resource management strategies to improve learning

#### Peer learning

In addition, our finding that peer learning significantly predicted first-year medical students’ learning outcomes corroborates those of previous research in non-medical face-to-face learning environments: that is, that students attained higher scores when asked to teach others about their learning material, as opposed to simply studying it. This effect may be due to the fact that when teaching others, students tend to think more meta-cognitively about the learning objectives that they want others to achieve; the teaching strategies they could use; and the questions they need to ask to check learners’ understanding.

Since students in a flipped-classroom environment are required to learn on their own before coming to class, peer learning could potentially help them decrease their learning anxiety, increase their learning engagement, and/or improve their understanding of the learning materials [26, 27]. It is thus especially important for medical educators to support peer learning in flipped-classroom environments, particularly given that within them, all foundational medical knowledge is acquired online, and students will need extra resources if they are to share the thinking load, ask and answer important questions, and effectively monitor their own progress.

At the present researchers’ instigation, the target medical school instituted four learning societies to support peer learning. Each consisted of a mixed group of medical students, clinical faculty, scientists, and social scientists. These societies intend to provide students with a favorable environment in which to communicate with peers and educators; and, as one student noted in the survey, “Seeing the same learning society fellow multiple times per week and forming a relationship with them, to have someone there that we are familiar with and can trust”, was an important affordance of learning in the medical school’s flipped-classroom environment.

#### Help seeking

With regard to the other strategy that positively predicted the sampled students’ learning outcomes - help-seeking from both instructors and peers - prior studies of flipped classrooms have indicated that students in such classrooms often find it hard to obtain timely help while studying independently [28]. Instant feedback via text messages or discussion boards could possibly help address this challenge. In any case, the present study’s finding of a positive correlation between help-seeking and learning outcomes in a flipped-classroom environment means that it is important for medical educators to provide better support for student help-seeking behavior. It is important to note however, that in the target medical school’s curriculum, a series of resources have already been provided to facilitate such behavior: for example, peer tutoring, in which upper-cohort students volunteer to tutor first-year students on a one-to-one basis, and the provision by learning specialists of consultation on how to use different self-directed learning strategies. In addition, platforms such as GroupMe and Facebook are used to provide virtual spaces in which students can instantly seek help from faculty and peers, and provide both academic and emotional support to one another.

### Insignificance of other cognitive and metacognitive strategies: does it mean they are not important?

Finally, although the present study findings imply that peer learning and help-seeking are both beneficial to the learning of early-stage medical students in a flipped-classroom environment, this should not be taken to mean that other SRL skills are not important or should not be used in such environments. As demonstrated in the above content-analysis results, the vocal students faced profound challenges to their SRL, including how to manage their time and how to deal with the overwhelming amount of information – echoing the results of one prior study, which found that some undergraduates in flipped-learning settings did not feel ready to take responsibility for their own learning [29]. Thus, more training and ongoing support will be needed to help students develop the SRL skills they will need to obtain the full benefits of the flipped classroom, and to help enable them to make a smooth transition from traditional lecture-focused learning [9]. For example, curriculum developers could provide concept maps or other graphic organizers to help students develop organization skills, or a learning coach could provide co-regulated learning opportunities with struggling students to share the cognitive and metacognitive thinking load [17].

One limitation of the present study is that it took place within a single medical school. Therefore, the findings might not be generalizable to other institutions. In addition, the sample size (*N* = 146) was comparatively small, and multiple regression with more than 10 independent variables, might result in insufficient statistical power. Further research could examine how students’ use of SRL strategies change over time throughout their learning in flipped classroom learning environments, and examine what factors affect students’ SRL strategy use.

Another limitation is the aptitude measure of SRL strategies within this study. Although MSLQ is considered as a valid instrument of students’ SRL strategies [30], a growing number of recent studies have called for event measures of SRL in both medical education [31] and non-medical education learning [32, 33]. Recent research has suggested that SRL is a dynamic process which should be captured as a real-time learning process, using measures such as SRL microanalysis, which consists of a structured interview protocol administrated immediately before, during, and after a specific learning task [34], a concurrent think-aloud protocol that asks participants to voice their thinking processes while working on a task [33], or eye-tracking data to record the paths of student learning [35]. Future research should consider utilizing methodologies that produce process data or multi-dimensional data to better understand how students learn.

## Conclusions

Although flipped classrooms are widely advocated as a new model for medical education [30], their effectiveness remains unclear [6]. The present study has therefore helped to fill a gap in the existing literature by exploring the relationship between self-regulation and learning outcomes among medical students using flipped classrooms. Our study found that peer learning and help-seeking were positively associated with student learning outcomes, for both first- and second-year medical students, respectively. The key implication of this for medical educators is that these resource management strategies are critically important for student success in flipped learning. Multiple formats of activities and resources for peer learning (such as learning communities and near-peer teaching) and help-seeking (such as tutoring, learning support, and text-message groups) could be provided to scaffold students’ SRL strategies development, which is of critical importance to learning success in flipped-classroom environments [8].

## Data Availability

The datasets generated and analyzed during the current study are available from the corresponding author on reasonable request.

## Abbreviations

SRL: Self-regulated learning
MSLQ: Motivational Strategies for Learning Questionnaire
NBME: National Board of Medical Examiners
CBSE: Comprehensive Basic Science Exam
